# Uncovering key regulatory pathways and prognostic biomarkers in the tumor microenvironment of high-grade serous ovarian cancer through single-cell RNA sequencing and experimental validation

**DOI:** 10.3389/fonc.2025.1591430

**Published:** 2025-05-09

**Authors:** Yue Li, Long Zhao, Ying Tian, Qianqian Zhou, Xia Liu, Shucai Yang, Jinfeng Xu, Chang Zou, Jinling Zhang, Hui Luo

**Affiliations:** ^1^ The First Affiliated Hospital of Shenzhen University, Shenzhen Second People’s Hospital, Shenzhen, Guangdong, China; ^2^ Department of Nuclear Medicine, Shenzhen People's Hospital (The First Affiliated Hospital, Southern University of Science and Technology, The Second Clinical Medical College, Jinan University), Shenzhen, Guangdong, China; ^3^ Department of Gynecology, Shenzhen People's Hospital (The First Affiliated Hospital, Southern University of Science and Technology, The Second Clinical Medical College, Jinan University), Shenzhen, Guangdong, China; ^4^ Department of Medical School, Southern University of Science and Technology, Shenzhen, China; ^5^ Department of Clinical Laboratory, Pingshan Hospital, Southern Medical University (Pingshan District People’s Hospital of Shenzhen), Shenzhen, China

**Keywords:** high-grade serous ovarian cancer (HGSOC), tumor microenvironment (TME), pleiotrophin (PTN) signaling, syndecan 4 (SDC4), single-cell RNA sequencing (scRNA-seq), experimental validation

## Abstract

**Background:**

High-grade serous ovarian cancer (HGSOC) is a leading cause of cancer-related deaths among women globally. This study aims to identify novel regulatory targets and signaling pathways that modulate the tumor microenvironment (TME) in HGSOC, focusing on the pleiotrophin (PTN) signaling pathway and syndecan 4 (SDC4) expression as potential biomarkers for prognosis.

**Methods:**

Bioinformatics analysis was conducted on single-cell RNA sequencing (scRNA-seq) data (GSE146026) of HGSOC to investigate the TME. The data were subjected to unsupervised clustering to classify cell types within the TME, revealing eight distinct clusters representing various cell types. Cell-cell interactions were analyzed using the CellChat tool. Additionally, TCGA datasets were used to validate the expression of SDC4 and its association with clinical outcomes. The functional enrichment of differentially expressed genes (DEGs) between high and low SDC4 expression groups was performed to uncover associated pathways. Experimental validation was carried out using quantitative real-time PCR (qRT-PCR) and Western blotting on ovarian cancer cell lines (OVCAR3 and SKOV3).

**Results:**

The unsupervised clustering analysis revealed eight major cell types: macrophages, fibroblasts, ovarian cancer cells, B cells, T cells, dendritic cells, and erythrocytes. CellChat analysis highlighted significant interactions between these cell types, suggesting a complex TME. Further exploration identified PTN signaling as a key regulator within the HGSOC TME. Validation using TCGA datasets revealed upregulation of SDC4 in ovarian cancer tissues, with high SDC4 expression correlating with shorter overall survival. DEGs between high and low SDC4 expression groups were linked to the PI3K-Akt and MAPK signaling pathways, cell junction organization, and focal adhesion. qRT-PCR validation confirmed a significant upregulation of SDC4 in OVCAR3 and SKOV3 ovarian cancer cell lines, with expression levels 3.8- to 4.2-fold higher than control cells (*p*<0.01), supporting the computational predictions.

**Conclusion:**

This study highlights the PTN signaling pathway as a potential therapeutic target in HGSOC and identifies SDC4 as a prognostic biomarker for poor patient outcomes. Our findings offer new insights into the molecular mechanisms governing the TME of HGSOC, although further investigation is needed to fully elucidate the functional role of SDC4 in ovarian cancer progression.

## Introduction

Ovarian cancer is one of the deadliest female malignant tumors, which can cause more than about 0.2 million women deaths annually in the world (1). The most common subtype of ovarian cancer is high-grade serous ovarian cancer (HGSOC), which accounts for about 80% of all ovarian cancer cases (2). As there is no obvious clinical symptoms during the early stage of HGSOC, more than 80% of patients with HGSOC were diagnosed at advanced stages (2). The patients with advanced-stage HGSOC had very poor survival outcomes, owing to the aggressiveness of this malignancy (3–6). Though chemotherapy, surgical resection and/or immunotherapy have been suggested as the main treatments, the recurrent rate of HGSOC is very high, and those patients had an overall 5-year survival of about 30% (3–6). Thus, exploring more effective therapies for patients with HGSOC is key for improved patient prognosis. HGSOC is characterized by two hallmarks. One is tumor heterogeneity, which is featured by the microenvironment of the tumor (7). Another hallmark is the epithelial-mesenchymal transition process, which plays a key role in regulating metastasis and chemoresistance of the HGSOC (8). Recently, studies have demonstrated that single-cell RNA sequencing (scRNA-seq) is very powerful in deciphering the tumor microenvironment (TME). For example, Deng et al. performed scRNA-seq analyses of primary HGSOC (HG_P) samples, metastatic HGSOC (HG_M) samples, and endometrioid carcinomas (EC) samples. The study demonstrated that ERBB2 and HOXB-AS3 genes were more amplified in metastasis tumors than in primary tumors, and revealed the TME of metastatic HGSOC (9). Xu et al., performed scRNA sequencing analyses of the tumors of 7 treatment-naïve patients with HGSOC at early or late stages and five age-matched non-malignant ovarian samples, and the results delineated an ecosystemic landscape of HGSOC at early or late stages with a focus on its heterogeneity with TME, and also identified a four-EMT gene model for prediction of HGSOC patient outcomes (8). Nath et al., profiled scRNA-seq transcriptomes of HGSOC tumors collected longitudinally during therapy, and revealed that HGSOC was driven by three archetypal phenotypes, defined as oncogenic states that describe the majority of the transcriptome variation (10). Recently, the data mining of scRNA-sequencing public data has been an important strategy for deciphering the TME in various types of cancers including HGSOC. For instance, Want et al., performed bioinformatics analysis of scRNA-seq data of HGSOC from GEO and identified several key prognostic biomarkers for HGSOC based on the stemness (11). In this study, we performed bioinformatics analysis of scRNA-seq data of high-grade serous ovarian cancer (HGSOC) from the GEO dataset (GSE146026). We analyzed cellular interactions within the TME of HGSOC to uncover key regulatory networks. Our analysis identified the pleiotrophin (PTN) signaling pathway as a significant network modulating the tumor cells of HGSOC. Furthermore, we discovered that syndecan 4 (SDC4) may act as an important modulator within the HGSOC TME. To validate these findings, experimental validation was performed using ovarian cancer cell lines (OVCAR3 and SKOV3), where quantitative real-time PCR (qRT-PCR) confirmed the upregulation of SDC4. Finally, the prognostic role of SDC4 in HGSOC was further evaluated using TCGA datasets, which demonstrated that high SDC4 expression is associated with poor patient prognosis in ovarian cancer.

## Materials and methods

Ethical approval was not required for this study as all experiments were conducted using standard, commercially available ovarian cancer cell lines OVCAR3 and SKOV3 and online data. All laboratory procedures adhered to standard protocols and were conducted in compliance with the laboratory standards of Shenzhen Second People’s Hospital.

### Data source collection and identification of cell types

Eight high-grade serous ovarian cancer (HGSOC) samples from six patients with single-cell RNA sequencing (scRNA-seq) data were obtained from the Gene Expression Omnibus (GEO) database (accession: GSE146026, (https://www.ncbi.nlm.nih.gov/geo/) (12). The sequencing data, generated using the 10X Genomics platform, were processed with the Seurat package (Version: 3.2.1) in R software (Version 3.6.3). Each sample dataset was integrated into the project, and quality control metrics, including mitochondrial gene expression percentage and unique molecular identifier (UMI) counts, were applied to filter out low-quality cells. Principal component analysis (PCA) was conducted to identify significant dimensions for clustering, followed by visualization using the t-distributed stochastic neighbor embedding (tSNE) method. Differentially expressed genes (DEGs) between cell clusters were determined using the “FindAllMarkers” function in Seurat, with thresholds of logFC ≥ 0.25, min.pct ≥ 0.25, and min.diff.pct ≥ 0.25.

### Cell-cell communication analysis

The R software package of CellChat (version 1.4.0) was used to infer and analyze intercellular cell-cell communication networks across all the cell types based on single-cell RNA sequencing (scRNA-seq) data (13). The ligand-receptor signaling pathways were inferred using the CellChat database, and only pathways with a *p*-value < 0.05 were considered statistically significant. The strength of cell-cell communication was further evaluated based on signaling pathway activity scores and cell-type-specific interactions.

### Survival analysis

The survival analysis was performed using the R package Survival (Version 3.4-0). The gene expression profiles of ovarian cancer patients were retrieved from The Cancer Genome Atlas (TCGA) (https://portal.gdc.cancer.gov/). Raw read counts were extracted from files with the suffix “htseq.counts.” Clinical data were also obtained, and patients were stratified into high-expression and low-expression groups based on the median expression levels of candidate genes. Kaplan-Meier survival curves were generated to assess the prognostic impact of gene expression, and statistical significance was determined using the log-rank test.

### GEPIA (GEPIA 2) datasets analysis

The Gene Expression Profiling Interactive Analysis (GEPIA) (http://gepia.cancer-pku.cn/) was used to verify the differentially expressed genes between ovarian cancers and normal tissues. GEPIA was a newly-developed tool for analyzing genetic differences based on TCGA datasets. GEPIA database was performed to identify the expression levels of SDC4.

### GO and KEGG enrichment analysis

Gene Ontology (GO) and Kyoto Encyclopedia of Genes and Genomes (KEGG) pathways on gene-set enrichment analysis were performed on the R package of clusterProfiler (v3.18.1) (14) with the following parameter: pvalueCutoff=0.05, pAdjustMethod=“BH”.

### Experimental validation

To validate key findings from the bioinformatics analysis, quantitative real-time PCR (qRT-PCR) were performed using independent ovarian cancer cell lines as IOSE80, a non-malignant ovarian epithelial cell line. The qRT-PCR validation, ovarian cancer cell lines OVCAR3 and SKOV3 were cultured in RPMI-1640 medium supplemented with 10% fetal bovine serum (FBS) and 1% penicillin-streptomycin under standard conditions (37°C with 5% CO_2_). Total RNA was extracted using TRIzol reagent (Invitrogen) according to the manufacturer’s protocol. RNA quality and concentration were assessed using a Nanodrop spectrophotometer (Thermo Fisher Scientific), and 1 µg of total RNA was reverse transcribed into complementary DNA (cDNA) using the High-Capacity cDNA Reverse Transcription Kit (Applied Biosystems). qRT-PCR was performed using SYBR Green (Thermo Fisher Scientific) on an ABI 7500 Fast Real-Time PCR system with gene-specific primers for SDC4 and other candidate genes. GAPDH was used as an internal reference, and relative gene expression levels were calculated using the ΔΔCt method.

## Results

### Bioinformatics analysis of GSE146026

**Figure 1:**
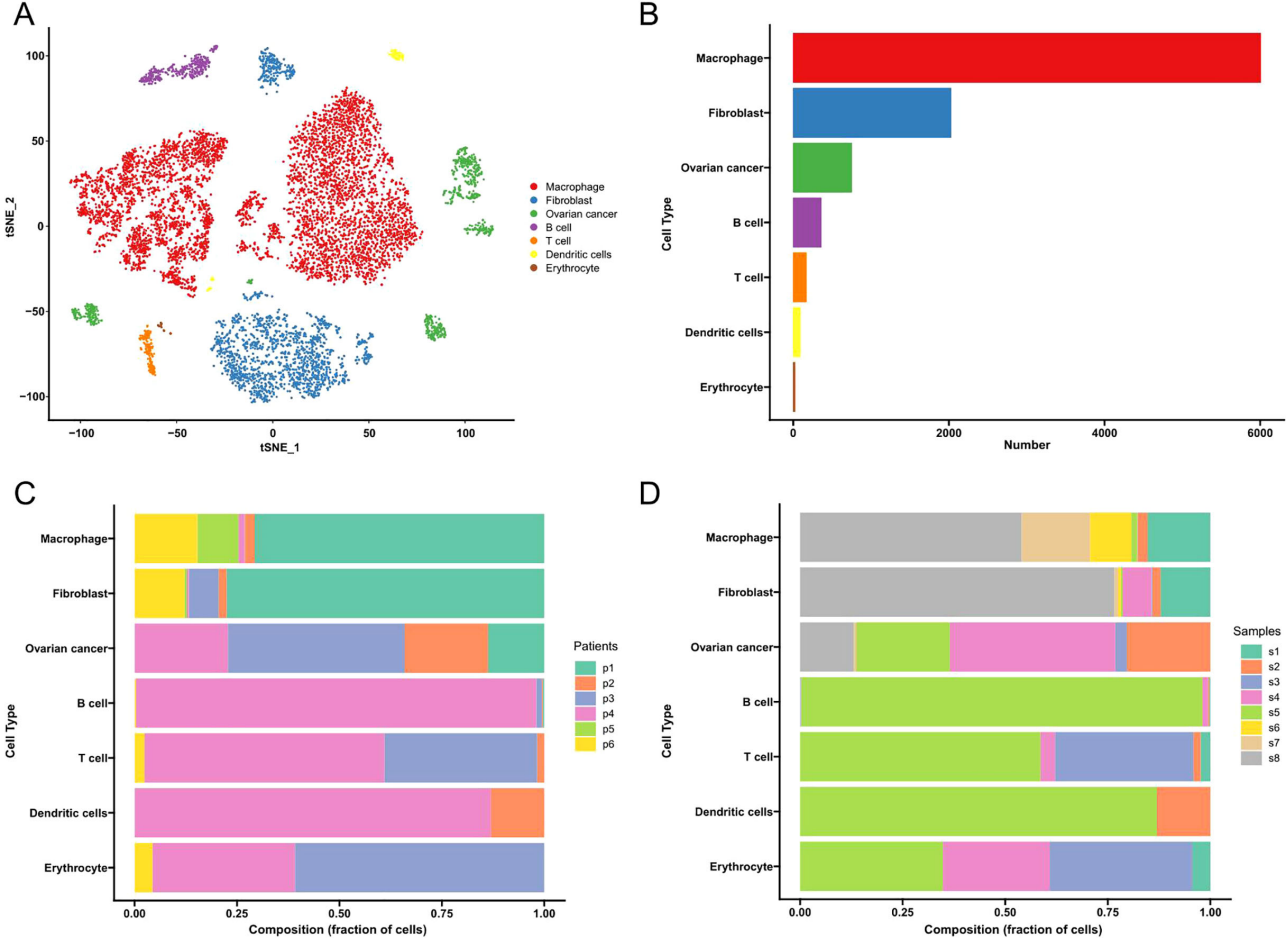
Cell types in HGSOC tissues delineated by scRNA-seq analysis. **(A)** The UMAP plot demonstrates the main cell types in HGSCO tissues. **(B)** The proportion of cell types in HGSOC tissues. **(C)** Cell type distribution from individual patients. **(D)** Cell type distribution from individual tissues.

We performed transcriptomic analysis using single-cell RNA sequencing (scRNA-seq) to investigate the tumor microenvironment (TME) of high-grade serous ovarian cancer (HGSOC). Unsupervised clustering of the scRNA-seq data resulted in the identification of eight distinct clusters, as shown in **Figure 1A**. These clusters correspond to individual cell types rather than patient or tissue identity, as demonstrated in **Figures 1B, C**. The identified cell types include macrophages, fibroblasts, ovarian cancer cells, B cells, T cells, dendritic cells, and erythrocytes. Notably, macrophages, fibroblasts, and ovarian cancer cells were the predominant cell types within the TME, as highlighted in **Figure 1D**. This clustering underscores the complexity of the cellular composition within the HGSOC microenvironment.

### Cell-cell interaction analysis

**Figure 2:**
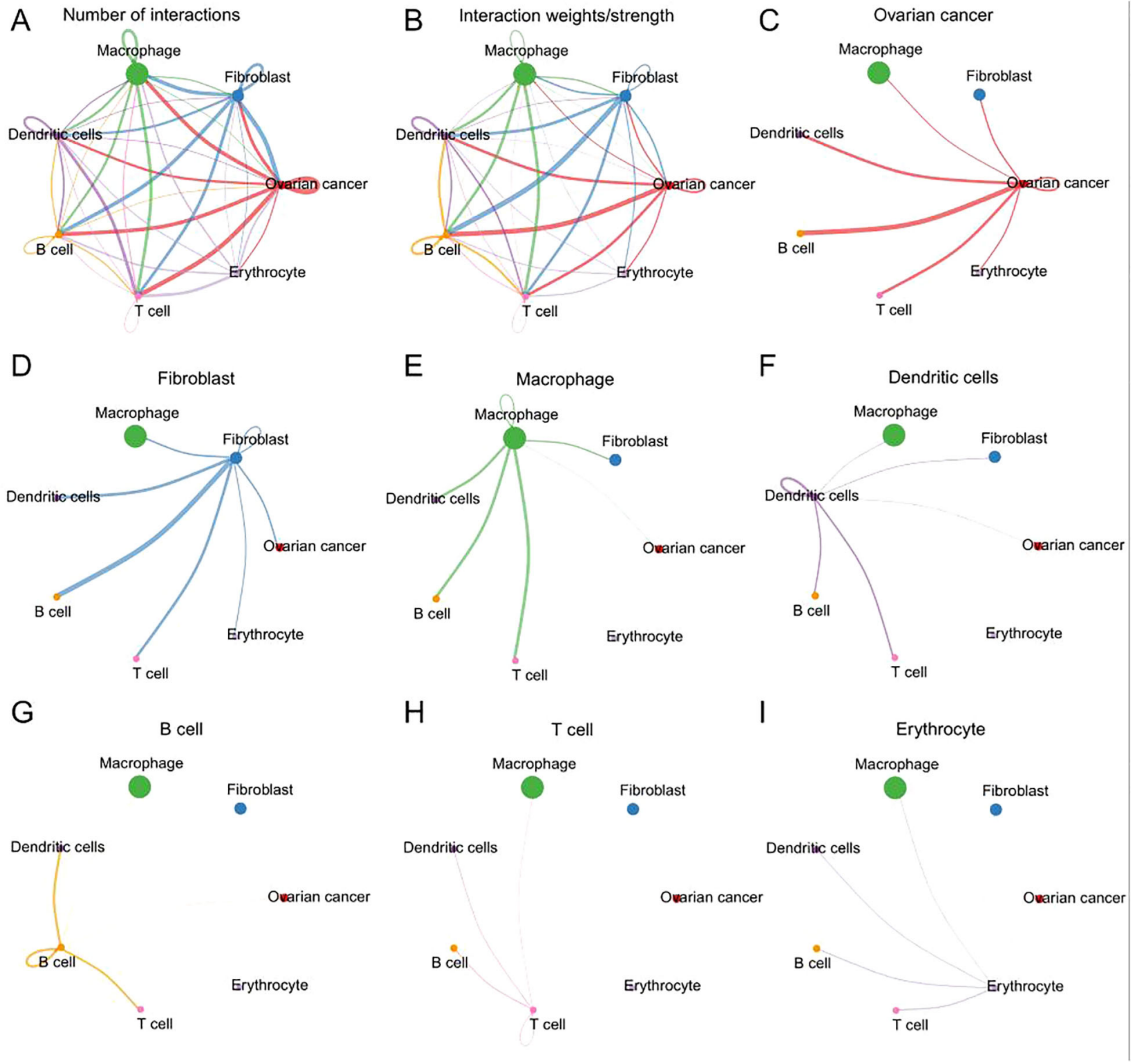
The interactions of ligand receptors among different cell types. **(A)** The number of ligand receptors interaction among all cell types. **(B)** The weights/strength of ligand receptors interaction among cell types. **(C)** The interaction of ligand receptors between ovarian cancer and other cell types. **(D)** The interaction of ligand receptors between fibroblast and other cell types. **(E)** The interaction of ligand receptors between macrophage and other cell types. **(F)** The interaction of ligand receptors between dendritic and other cell types. **(G)** The interaction of ligand receptors between B cell and other cell types. **(H)** The interaction of ligand receptors between T cell and other cell types. **(I)** The interaction of ligand receptors between erythrocytes and other cell types.

The numbers and weights of ligand receptors were evaluated using the CellChat analysis. In terms of the number of interactions, the “macrophage” showed high number of interactions with “fibroblast”, “ovarian cancer”, and vice versa; the “fibroblast” showed high number of interactions with “ovarian cancer”, “dendritic cells”, “B cell”, “T cell “and vice versa; the “ovarian cancer” showed high number of interactions with “T cell”, “B cell” and vice versa (**Figure 2A**). The similar interaction patterns between different cell types were shown in interaction weights/strength (**Figure 2B**). The detailed interactions for each type of cell were further illustrated in **Figures 2C–I**.

### Analysis of outgoing and incoming interaction strength among cell types in PTN signaling

**Figure 3:**
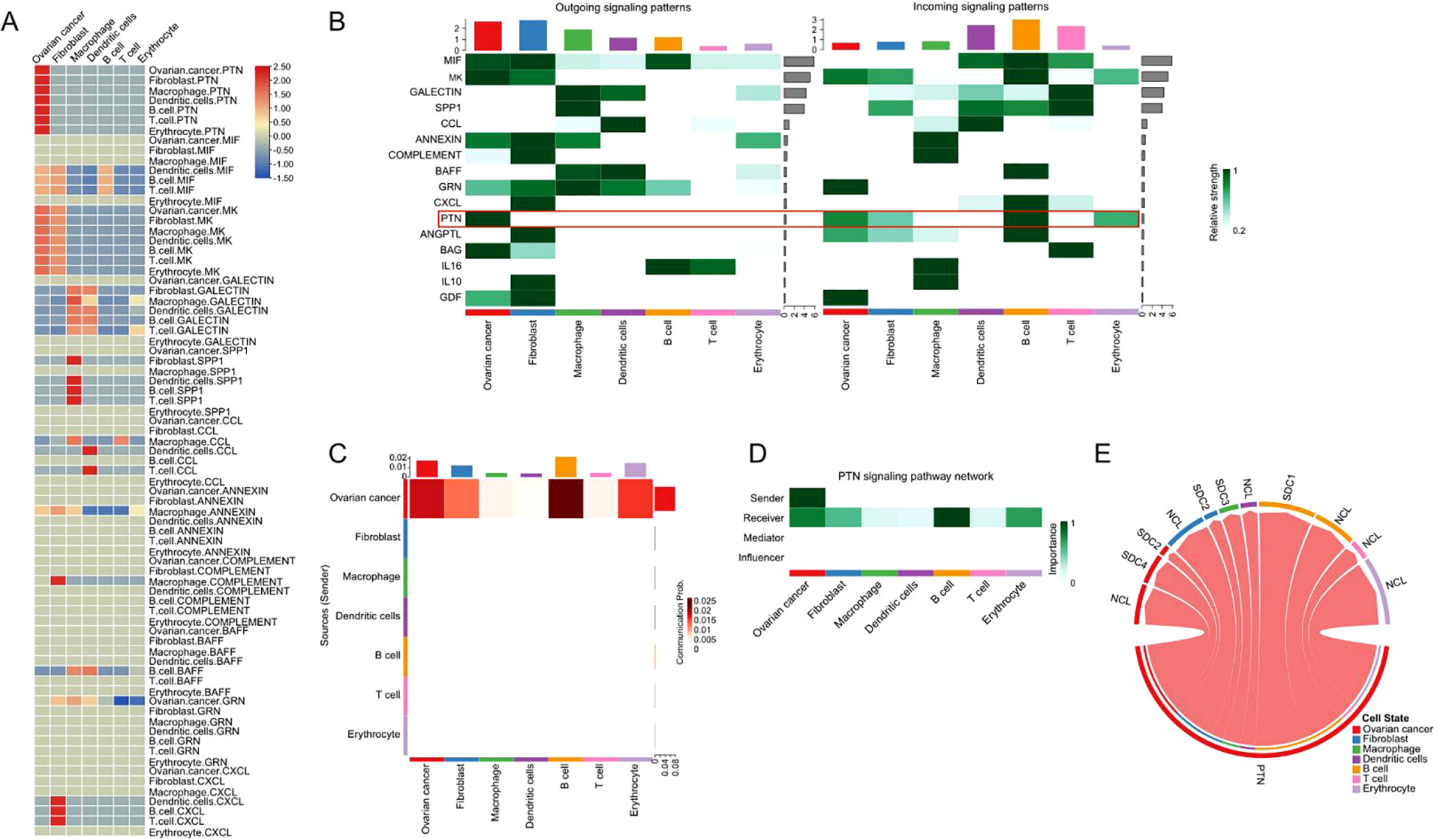
The outgoing and incoming interaction strength among all cell types. **(A)** The heatmap illustrates the signaling strength among the cellular interactions for all cell types. **(B)** The outgoing and incoming interaction strength for different signaling pathways among all cell types. **(C)** The heatmap illustrates the PTN signaling strength among all cell types. **(D)** The dominant senders, receivers, mediators and influencers in the intercellular PTN signal communication network. **(E)** The chord diagram illustrates the ligand receptor interactions of PTN signaling in all types of cells.

We analyzed the outgoing and incoming interaction strengths of signaling pathways within the tumor microenvironment (TME) of high-grade serous ovarian cancer (HGSOC) using scRNA-seq data. The heatmap in **Figure 3A** reveals significant ligand-receptor interactions, with the PTN signaling pathway showing the strongest correlation. Outgoing signals were predominantly contributed by “ovarian cancer,” “fibroblast,” and “macrophage” cell types, while “B cells,” “T cells,” and “dendritic cells” were the main contributors to incoming signaling. Notably, PTN is the only signaling pathway originating from “ovarian cancer” and received by all other cell types (**Figure 3B**). Further analysis (**Figure 3C**) demonstrated that PTN signaling was particularly strong in “B cells,” “ovarian cancer,” “erythrocytes,” and “fibroblasts,” suggesting their pivotal roles in the TME. The network analysis in **Figure 3D** highlighted “ovarian cancer” as the dominant sender of PTN signals, with “B cells” identified as the most significant receivers. Finally, the ligand-receptor interactions of PTN signaling, illustrated in **Figure 3E**, emphasize its crucial role in regulating intercellular communication within the HGSOC TME, highlighting PTN as a central modulator of cellular interactions in ovarian cancer.

### Analysis of gene expressions in the PTN signaling among different cell types

**Figure 4:**
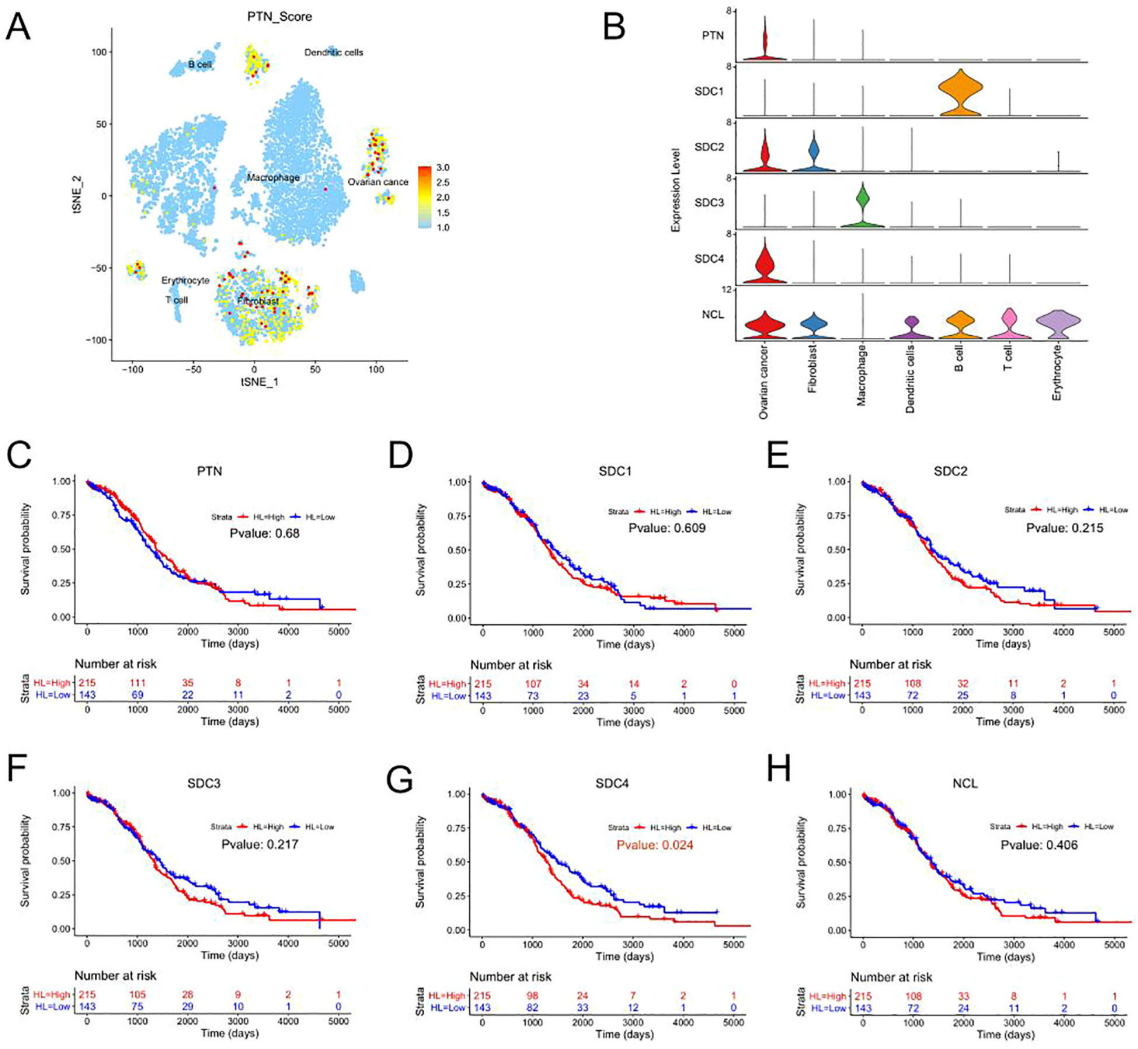
Prognostic prediction of genes in the PTN signaling pathway. **(A)** The UMAP plot demonstrates expression levels of PTN signaling-associated genes in the 8 cell types. **(B)** The violin plots illustrate the expression levels of genes from PTN signaling pathway in the 8 cell types. **(C-H)** The association between the expression levels of **(C)** PTN, **(D)** SCD1, **(E)** SDC2, **(F)** SDC3, **(G)** SDC4, **(H)** NCL and overall survival of patients with ovarian cancer was shown as Kaplan-Meier plot.

The expression levels of genes in the PTN signaling was further analyzed. As shown in **Figure 4A**, the genes in the PTN signaling were mainly expressed in the cell types including “fibroblast” and “ovarian cancer”. In addition, the expression of genes from PTN signaling in different cell types was illustrated by a violin plot (**Figure 4B**). Furthermore, the prognostic role of genes in the PTN signaling was evaluated by using TGCA datasets. As shown in **Figures 4C–H**. PTN, SDC1, SDC2, SDC3 and NCL expression was not associated with the overall survival of patients with ovarian cancer; while high expression of SDC4 was significantly correlated with shorter overall survival of patients with ovarian cancer.

### The functional role of SDC4 in ovarian cancer

**Figure 5:**
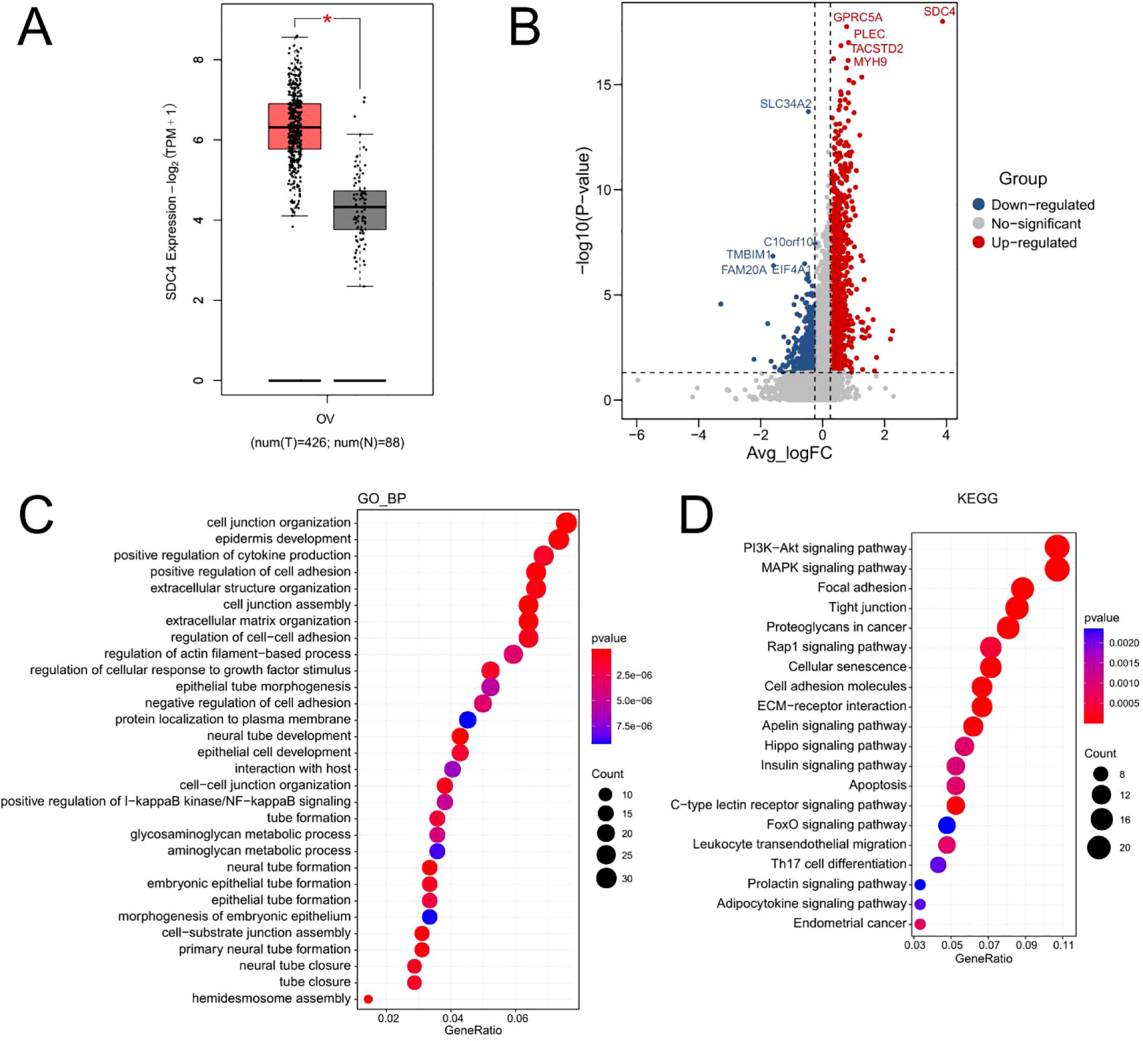
The functional role of SDC4 in ovarian cancer. **(A)** The expression of SDC4 in normal and cancerous ovarian tissues was analyzed using the GEPIA tool. **(B)** The differentially expressed genes between SDC4 high expression and low expression groups were compared in the ovarian cancer cells, and the DEGs were shown as a volcano plot. **(C)** The GO_biological process enrichment analysis of DEGs. **(D)** The KEGG enrichment analysis of DEGs.

The expression of SDC4 in ovarian cancer tissues was further analyzed using GEPIA tool. As shown in **Figure 5A**, the expression level of SDC4 was significantly higher in the ovarian cancer tissues than that in the normal ovarian tissues. The ovarian cancer cell type was classified into SDC4 high expression and SDC4 low expression groups. The differentially expressed genes between SDC high expression and low expression groups were compared in the ovarian cancer cells, and the DEGs were shown as a volcano plot (**Figure 5B**). Furthermore, the DEGs were subjected to functional enrichment analysis. As shown **Figure 5C**, the GO enrichment analysis showed that the DEGs was mainly enriched in the GO_biological process terms including “cell junction organization”, “epidermis development”, “positive regulation of cytokine production” and so on (**Figure 5C**). The KEGG enrichment analysis revealed that the DEGs were mainly enriched in the KEGG pathways including “PI3K-Akt signaling pathway”, “MAPK signaling pathway”, “Focal adhesion”, “Tight junction” and so on (**Figure 5D**).

We applied a threshold of logFC ≥ 0.25 and min.pct ≥ 0.25 to identify differentially expressed genes. These criteria were chosen to ensure the inclusion of genes with moderate but potentially biologically significant expression changes, which might be overlooked with more stringent cutoffs. Given that scRNA-seq data often exhibit high dropout rates and technical variability, slightly relaxed thresholds can enhance the detection of meaningful biological signals while maintaining statistical rigor. However, to further validate our findings, we performed functional enrichment analysis on the identified DEGs, confirming their involvement in key pathways such as PI3K-Akt, MAPK signaling, and focal adhesion supporting their potential role in ovarian cancer progression.

### *Ex-vivo* validation

**Figure 6:**
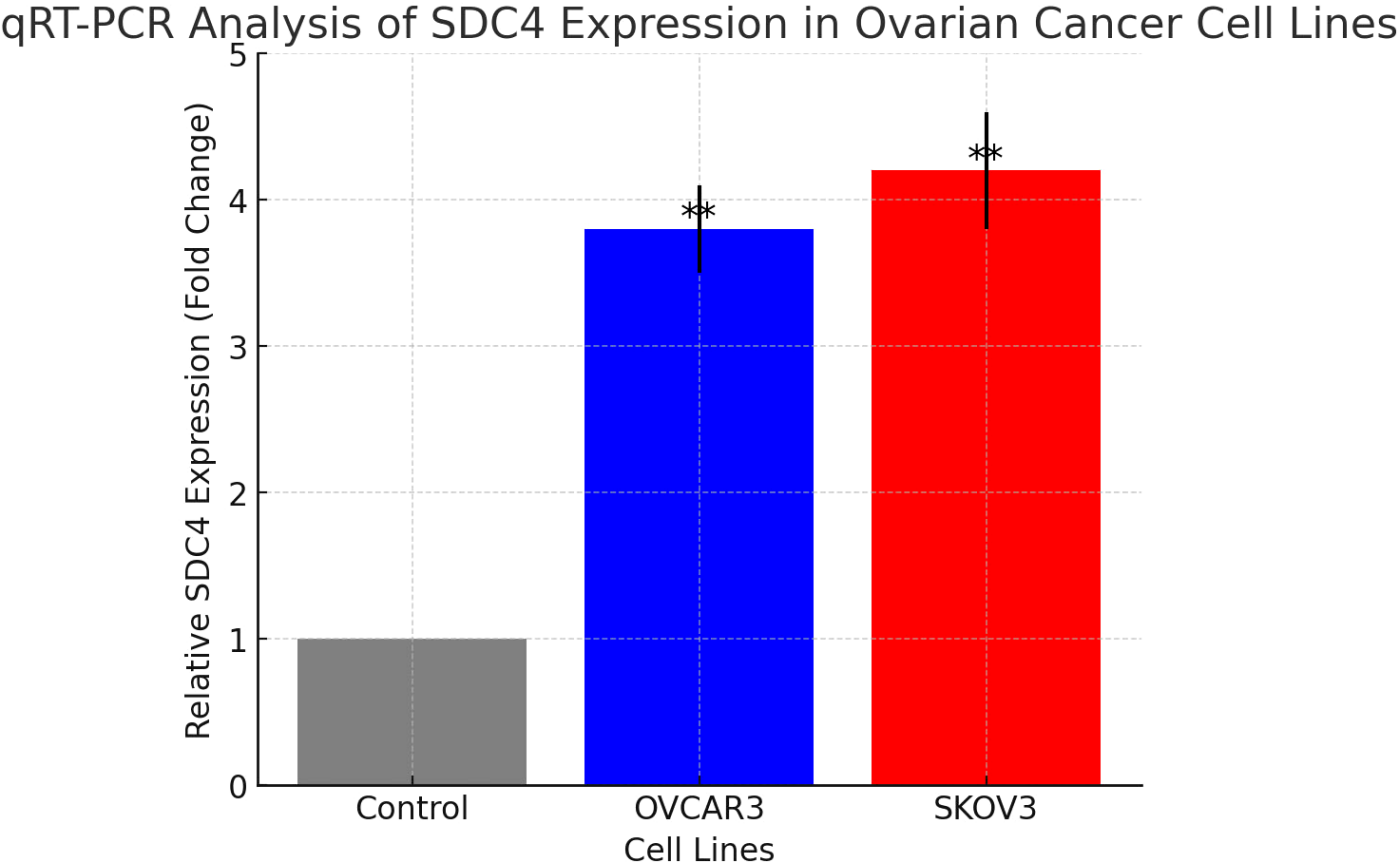
Relative expression of SDC4 in ovarian cancer cell lines OVCAR3 and SKOV3 compared to control, measured by qRT-PCR. **: pvalue < 0.01.

qRT-PCR analysis confirmed a significant upregulation of SDC4 in ovarian cancer cell lines compared to control samples. The expression levels of SDC4 were markedly higher in OVCAR3 and SKOV3 cells, consistent with the bioinformatics predictions. OVCAR3 exhibited a 3.8-fold increase, while SKOV3 showed a 4.2-fold increase in SDC4 expression, relative to the control (*p* < 0.01) (**Figure 6**). These results further support the computational findings, indicating a potential role of SDC4 in ovarian cancer progression.

## Discussion

With the advancement of high-throughput technology, the scRNA-seq has recently become a powerful tool to decipher the TME (15–17). The present study performed the bioinformatics analysis of scRNA-seq dataset (GSE146026) for HGSOC tissues. Our analysis identified 8 clusters of cells, with “macrophages”, “fibroblast” and “ovarian cancer” being the main cell types. The CellChat analysis revealed the close interaction between different cell types. Further analysis revealed that PTN signaling is a player in the regulation of HGSOC TME. By using TGCA datasets, we found that SDC4 was up-regulated in the ovarian cancer tissues and high expression of SDC4 in ovarian cancer tissues was associated with shorter overall survival of patients with ovarian cancer. The functional enrichment analysis of DEGs between high and low SDC4 expression groups revealed that the DEGs were associated with cell junction organization, PI3K-Akt signaling pathway, MAPK signaling pathway and focal adhesion. Our analysis underpinned the important role of PTN signaling in the TME of HGSOC.

PTN is a heparin-binding growth factor that is highly expressed in certain solid cancers, such as in breast and lung cancers (18). PTN activates its cell surface receptors, regulating multiple functions including cell adhesion, cell migration, cell proliferation and cytoskeletal stability (19, 20). The role of PTN signaling has been well-documented in tumor biology. Shi et al., showed that tumor-associated macrophages secrete PTN to promote PTPRZ1 signaling in glioblastoma stem cells for tumor growth (21). Feng et al., demonstrated that lung cancer cell migration can be hampered via repressing growth factor PTN/RPTP β/ζ signaling by menin (22). Activation of PTN signaling could promote perineural invasion in the pancreatic cancer (23). Kong et al., found that PTN was a potential colorectal cancer prognostic factor that promoted VEGF expression and induced angiogenesis in the colorectal cancer (24). In ovarian cancer studies, PTN was expressed, produced, and secreted in a panel of EOC cell lines. PTN levels in serous ovarian tumor tissues were on average 3.5-fold higher relative to normal tissue and PTN is detectable in serum samples of patients with EOC. The 3.8- to 4.2-fold increase in SDC4 expression was observed in ovarian cancer tissues compared to normal ovarian tissues, serving as the baseline for this comparison. These findings underpinned that PTN and its signaling components may be of significance in the pathogenesis of epithelial ovarian cancer (19). In our analysis, we revealed that PTN is one of major driven signaling pathway in ovarian cancer cells that affect the other cell types in the TME, suggesting the importance of PTN signaling in the TME of HGSOC. Among these genes from PTN signaling, we further explored the relationship between the expression of these genes and the overall survival of patients with ovarian cancer. We found that high expression of SDC4 was associated with shorter overall survival of patients with ovarian cancer. SDC4 is an important member of SDCs family. SDC4 core protein mainly contains extracellular domain, transmembrane (TM) domain, and cytoplasmic (CP) domain (25–28). Extracellular domain allows SDC4 interactions with extracellular matrix proteins through its heparan sulfate (HS) chain and SDC4 core also engage in protein-protein interactions directly. Syndecan-4 (SDC4) functions as a major endogenous membrane-associated receptor and widely regulates cytoskeleton, cell adhesion, and cell migration in human tumorigenesis (29). For examples, Chen et al., showed that SDC4 gene silencing could promote human papillary thyroid carcinoma cell apoptosis and inhibit epithelial mesenchymal transition via Wnt/β-catenin pathway (26). High expression of SDC-4 was found to be related to clinicopathological features and poor prognosis of pancreatic adenocarcinoma (30). In ovarian cancer studies, Kim et al., showed that CCL5, a chemotactic ligand, was enriched in immune cells (T cells and NK cells) and mediated ovarian cancer cell survival in the ascites, through SDC4. Moreover, SDC4 expression correlated with poor overall survival in ovarian cancer patients (31). Given that high SDC4 expression correlates with poor prognosis, it may serve as a potential biomarker for patient stratification in HGSOC. Patients with elevated SDC4 expression could be prioritized for more aggressive therapeutic strategies, including closer monitoring or targeted interventions. Furthermore, as SDC4 is involved in cell adhesion and migration, it might serve as a therapeutic target in combination with existing treatments. Future studies should investigate whether SDC4 expression levels can predict response to chemotherapy or targeted therapies, further enhancing its clinical utility in personalized medicine. The qRT-PCR validation further corroborated our bioinformatics predictions, showing a significant upregulation of SDC4 expression in ovarian cancer cell lines, particularly in OVCAR3 and SKOV3, relative to control cells. These results provide experimental evidence supporting the association between SDC4 overexpression and the molecular mechanisms underlying ovarian cancer progression. Furthermore, our study suggests that SDC4 may contribute to the progression of HGSOC, possibly by modulating cell interactions within the TME. Despite the valuable insights provided by our bioinformatics and experimental validation, this study has some limitations. First, the scRNA-seq data used in the analysis were derived from a publicly available dataset, which may not fully represent the complexity of HGSOC across different patient cohorts. Additionally, while our findings indicate a correlation between SDC4 expression and prognosis, the functional validation of SDC4’s role in HGSOC progression requires further *in vitro* and *in vivo* studies, such as knockdown or overeDEGs expression experiments, to establish a definitive causal relationship. These experiments could help determine whether SDC4 directly contributes to tumor growth, metastasis, or chemoresistance.

## Conclusion

Present study underscores the critical role of PTN signaling in shaping the tumor microenvironment (TME) of high-grade serous ovarian cancer (HGSOC). Through comprehensive bioinformatics analysis, we identified PTN signaling as a key mediator of intercellular communication within the TME, influencing various cellular interactions that contribute to tumor progression. Notably, we observed that high expression of SDC4, a critical component of PTN signaling, is significantly associated with poor prognosis in ovarian cancer patients, highlighting its potential as a prognostic biomarker. Functional enrichment analysis of differentially expressed genes between high and low SDC4 expression groups revealed pathways related to cell junction organization, PI3K-Akt signaling, MAPK signaling, and focal adhesion, suggesting that SDC4 may play a pivotal role in ovarian cancer cell adhesion, migration, and overall tumor progression. These findings provide valuable insights into the molecular mechanisms driving HGSOC and highlight potential therapeutic targets within the PTN-SDC4 signaling axis. However, further functional validation and clinical studies are required to establish the definitive role of SDC4 in HGSOC progression and its potential utility as a therapeutic target or prognostic marker.

## Data Availability

Thank you for the comment. We have included a dedicated “Data Availability” section in the article to clarify how the data used in the study can be accessed. The scRNA-seq dataset (GSE146026) is publicly available through the Gene Expression Omnibus (GEO) repository. Additionally, all other relevant data, including the results of the bioinformatics analyses and qRT-PCR validation, can be made available upon reasonable request to the corresponding authors.
